# Updated list of *Anopheles* species (Diptera: Culicidae) by country in the Afrotropical Region and associated islands

**DOI:** 10.11646/zootaxa.4747.3.1

**Published:** 2020-03-04

**Authors:** Seth R. Irish, David Kyalo, Robert W. Snow, Maureen Coetzee

**Affiliations:** 1 U.S. President’s Malaria Initiative and Entomology Branch, Division of Parasitic Diseases and Malaria, Center for Global Health, Centers for Disease Control and Prevention, 1600 Clifton Road NE, Atlanta, GA 30329, USA; 2 Population Health Unit, Kenya Medical Research Institute—Wellcome Trust Research Programme, P.O. Box 43640-00100, Nairobi, Kenya; 3 Centre for Tropical Medicine and Global Health, Nuffield Department of Clinical Medicine, University of Oxford, Oxford, UK; 4 Wits Research Institute for Malaria, School of Pathology, Faculty of Health Sciences, University of the Witwatersrand and Centre for Emerging Zoonotic & Parasitic Diseases, National Institute for Communicable Diseases, Johannesburg, South Africa

**Keywords:** Africa, *Anopheles*, mosquitoes, inventory

## Abstract

The distributions of the Afrotropical *Anopheles* mosquitoes were first summarized in 1938. In 2017, an extensive geocoded inventory was published for 48 sub-Saharan African countries, including information such as sampling methods, collection dates, geographic co-ordinates and the literature consulted to produce the database. Using the information from the 2017 inventory, earlier distribution lists, museum collections and publications since 2016, this paper presents an updated, simplified list of *Anopheles* species by mainland countries and associated Afrotropical islands, with comments where applicable. It is intended as a supplement to the 2017 geo-coded inventory.

## Introduction

At the end of the 19th century, the *Anopheles* mosquitoes of the world became the focus of intense research after they were implicated in the transmission of malaria parasites (Ross 1910). Since then, lists of species recorded for sub-Saharan African countries were published by Evans (1938), Edwards (1941) and de Meillon (1947), with the country lists in Gillies & de Meillon (1968) being the most comprehensive at that time. A supplement to Gillies & de Meillon was subsequently published by Gillies & Coetzee (1987), which provided updated country occurrence records for some species, including newly described species. An interactive list and identification key for *Anopheles* of the Afrotropical Region was produced in 1998 (Hervy *et al.* 1998).

Although maps of dominant vector species were produced more recently (Sinka *et al.* 2010, 2012), these were limited to species involved in malaria transmission. It has taken almost 50 years for complete country lists to be updated. In 2017, Kyalo and co-workers produced a geo-coded inventory of *Anopheles* species recorded for 48 countries in sub-Saharan Africa, covering almost 120 years of work on this important group of insects (Kyalo *et al.* 2017). They also produced a freely accessible database of species by country that includes collection information, collection dates, geographic co-ordinates and reference sources that provide historic information on mosquito surveys conducted in Afrotropical countries over the years.

This present paper provides an update of the lists given in Table 3 of Kyalo *et al.* (2017), with some deletions and some additions of species to countries and notes on the rationale behind the amendments.

## Methods

The list of all the species present in each country, from Table 3 in Kyalo *et al.* (2017), was compared with the Kyalo *et al.* online database, the VectorMap lists provided by the Walter Reed Biosystematics Unit (WRBU) based in the Smithsonian Institution (http://vectormap.si.edu), records from Gillies & de Meillon (1968) and Gillies & Coetzee (1987) and the database of the Institut de Recherche pour le Développement (IRD) at Montpellier (https://arim.ird.fr/#recherches/index/specimens/routage:home). Species listed in the IRD database but not in the published literature, that are clearly way out of their normal distributions, have not been included in the country lists and require confirmation.

In addition to records from the collections of the National Institute for Communicable Diseases, Johannesburg, South Africa, noted by one of the authors (MC), a visit was made to the Natural History Museum in London, UK by another of the authors (SRI) in January 2019, and records noted during the visit are also included here.

One of the possible uses of these lists is the development of country-specific identification keys. For this reason, both malaria vectors and non-vectors have been included.

## Results

Each country list (Appendix) is followed by relevant comments regarding species additions, deletions or points of interest, and references to these are provided.

The *Anopheles* fauna of mainland Tanzania and Zanzibar are presented separately. In addition to the countries in Kyalo *et al.* (2017), *Anopheles* records for Mauritius, La Réunion and Lesotho are presented. No records were found for St. Helena, and despite an early report of *Anopheles gambiae s.l.* in the Seychelles, it appears that no *Anopheles* are present there (Robert *et al.* 2011; Le Goff *et al.* 2012).

Table 1 provides a list of all the currently recognised species by subgenus, series and authorship. An Excel file providing a single record for each species present in each country can be found at https://doi.org/10.7910/DVN/ PHGADL.

## Discussion

The species listed per country in Gillies & de Meillon (1968) are not always accompanied by references to published records. This is because M. T. Gillies personally studied the collections in the British, French, Belgian and South African museums to record species deposited in those collections that had never been documented in the published literature. Thus, for example, the inclusion of *Anopheles cydippis* de Meillon, *An. walravensi* Edwards and *An. ziemanni* Grünberg in the Botswana list would all have been based on observations from the collections in the South African Institute for Medical Research in Johannesburg (now the National Institute for Communicable Diseases), and reference to their presence would therefore be Gillies & de Meillon (1968). Further information on the museum specimens examined by Gillies (date of collection, location, collector, etc.) would necessitate a visit to the relevant museums as these details are not provided in Gillies & de Meillon (1968).

Species name changes, border changes and splitting one species into multiple species make maintaining these lists challenging. The use of chromosomal and molecular methods is increasingly being used to understand mosquito taxonomy. The adverb “*sensu lato*”, or the abbreviation “*s.l.*”, has been used for *Anopheles gambiae* Giles and *An. funestus* Giles where genetic/molecular species identification was not carried out. In particular, the listing of *An. gambiae s.l.* denotes that no differentiation was made in the past 30 years between *An. gambiae sensu stricto* (*s.s.*) and *An. coluzzii* Coetzee & Wilkerson (previously S and M molecular forms and Savanna and Mopti chromosomal forms, respectively) (Coetzee *et al.* 2013). Subspecies names are not included in the current list, only the nominal species is given.

## Supplementary Material

Appendix

## Figures and Tables

**Table 1:** *Anopheles* species of the Afrotropical Region and associated islands.

Subgenus	Species and authorship	Series
*Anopheles*	*caliginosus* de Meillon, 1943	Myzorhynchus
*Anopheles*	*concolor* Edwards, 1938	Anopheles
*Anopheles*	*coustani* Laveran, 1900	Myzorhynchus
*Anopheles*	*crypticus* Coetzee, 1995	Myzorhynchus
*Anopheles*	*fuscicolor* van Someren, 1947	Myzorhynchus
*Anopheles*	*namibiensis* Coetzee, 1984	Myzorhynchus
*Anopheles*	*obscurus* (Grünberg, 1905)	Myzorhynchus
*Anopheles*	*paludis* Theobald, 1900	Myzorhynchus
*Anopheles*	*symesi* Edwards, 1928	Myzorhynchus
*Anopheles*	*tenebrosus* Dönitz, 1902	Myzorhynchus
*Anopheles*	*ziemanni* Grünberg, 1902	Myzorhynchus
*Cellia*	*amharicus* Hunt, Wilkerson & Coetzee, 2013	Pyretophorus
*Cellia*	*arabiensis* Patton, 1905	Pyretophorus
*Cellia*	*ardensis* (Theobald, 1905)	Neomyzomyia
*Cellia*	*argenteolobatus* (Gough, 1910)	Cellia
*Cellia*	*aruni* Sobti, 1968	Myzomyia
*Cellia*	*austenii* (Theobald, 1905)	Myzomyia
*Cellia*	*azaniae* Bailly-Choumara, 1960	Myzomyia
*Cellia*	*azevedoi* Ribeiro, 1969	Paramyzomyia
*Cellia*	*barberellus* Evans, 1932	Myzomyia
*Cellia*	*berghei* Vincke & Leleup, 1949	Myzomyia
*Cellia*	*bervoetsi* D’Haenens, 1961	Myzomyia
*Cellia*	*brohieri* Edwards, 1929	Myzomyia
*Cellia*	*brucei* Service, 1960	Myzomyia
*Cellia*	*brumpti* Hamon & Rickenbach, 1955	Cellia
*Cellia*	*brunnipes* (Theobald, 1910)	Myzomyia
*Cellia*	*buxtoni* Service, 1958	Neomyzomyia
*Cellia*	*bwambae* White, 1985	Pyretophorus
*Cellia*	*cameroni* de Meillon & Evans, 1935	Neomyzomyia
*Cellia*	*carnevalei* Brunhes, Le Goff & Geoffroy, 1999	Neomyzomyia
*Cellia*	*caroni* Adam, 1961	Neomyzomyia
*Cellia*	*carteri* Evans & de Meillon, 1933	Myzomyia
*Cellia*	*christyi* (Newstead & Carter, 1911)	Pyretophorus
*Cellia*	*cinctus* (Newstead & Carter, 1910)	Neomyzomyia
*Cellia*	*cinereus* Theobald, 1901	Paramyzomyia
*Cellia*	*coluzzii* Coetzee & Wilkerson, 2013	Pyretophorus
*Cellia*	*comorensis* Brunhes, Le Goff & Geoffroy, 1997	Pyretophorus
*Cellia*	*confusus* Evans & Leeson, 1935	Myzomyia
*Cellia*	*cristipalpis* Service, 1977	Cellia
*Cellia*	*culicifacies* Giles, 1901	Myzomyia
*Cellia*	*cydippis* de Meillon, 1931	Cellia
*Cellia*	*dancalicus* Corradetti, 1939	Neocellia
*Cellia*	*daudi* Coluzzi, 1958	Pyretophorus
*Cellia*	*deemingi* Service, 1970	Neomyzomyia
*Cellia*	*demeilloni* Evans, 1933	Myzomyia
*Cellia*	*distinctus* (Newstead & Carter, 1911)	Myzomyia
*Cellia*	*domicolus* Edwards, 1916	Myzomyia
*Cellia*	*dthali* Patton, 1905	Myzomyia
*Cellia*	*dualaensis* Brunhes, Le Goff & Geoffroy, 1999	Neomyzomyia
*Cellia*	*dureni* Edwards, 1938	Neomyzomyia
*Cellia*	*eouzani* Brunhes, Le Goff & Boussès, 2003	Neomyzomyia
*Cellia*	*erepens* Gillies, 1958	Myzomyia
*Cellia*	*erythraeus* Corradetti, 1939	Myzomyia
*Cellia*	*ethiopicus* Gillies & Coetzee, 1987	Myzomyia
*Cellia*	*faini* Leleup, 1952	Neomyzomyia
*Cellia*	*flavicosta* Edwards, 1911	Myzomyia
*Cellia*	*fontenillei* Barrón, Paupy, Rahola, Akone-Ella, Ngangue, Wilson-Bahun, Pombi, Kengne, Costantini, Simard, González & Ayala, 2019	Pyretophorus
*Cellia*	*fontinalis* Gillies & de Meillon, 1968	Myzomyia
*Cellia*	*freetownensis* Evans, 1925	Myzomyia
*Cellia*	*funestus* Giles, 1900	Myzomyia
*Cellia*	*funestus-*like species (see Spillings *et al*., 2009)	Myzomyia
*Cellia*	*fuscivenosus* Leeson, 1930	Myzomyia
*Cellia*	*gabonensis* Rahola, Makanga & Paupy, 2014	Myzomyia
*Cellia*	*gambiae* Giles, 1902	Pyretophorus
*Cellia*	*garnhami* Edwards, 1930	Myzomyia
*Cellia*	*gibbinsi* Evans, 1935	Myzomyia
*Cellia*	*grassei* Grjebine, 1953	Neomyzomyia
*Cellia*	*grenieri* Grjebine, 1964	Neomyzomyia
*Cellia*	*griveaudi* Grjebine, 1960	Neomyzomyia
*Cellia*	*hamoni* Adam, 1962	Neomyzomyia
*Cellia*	*hancocki* Edwards, 1929	Myzomyia
*Cellia*	*hargreavesi* Evans, 1927	Myzomyia
*Cellia*	*harperi* Evans, 1936	Myzomyia
*Cellia*	*hervyi* Brunhes, Le Goff & Geoffroy, 1999	Neocellia
*Cellia*	*hughi* Lambert & Coetzee, 1982	Myzomyia
*Cellia*	*jebudensis* Froud, 1944	Neomyzomyia
*Cellia*	*keniensis* Evans, 1931	Myzomyia
*Cellia*	*kingi* Christophers, 1923	Neomyzomyia
*Cellia*	*kosiensis* Coetzee, Segerman & Hunt, 1987	Myzomyia
*Cellia*	*lacani* Grjebine, 1953	Neomyzomyia
*Cellia*	*leesoni* Evans, 1931	Myzomyia
*Cellia*	*letabensis* Lambert & Coetzee, 1982	Myzomyia
*Cellia*	*listeri* de Meillon, 1931	Paramyzomyia
*Cellia*	*lloreti* Gil Collado, 1935	Myzomyia
*Cellia*	*longipalpis* (Theobald, 1903)	Myzomyia
*Cellia*	*lounibosi* Gillies & Coetzee, 1987	Neomyzomyia
*Cellia*	*lovettae* Evans, 1934	Neomyzomyia
*Cellia*	*machardyi* Edwards, 1930	Neomyzomyia
*Cellia*	*maculipalpis* Giles, 1902	Neocellia
*Cellia*	*maliensis* Bailly-Choumara & Adam, 1959	Neomyzomyia
*Cellia*	*marshallii* (Theobald, 1903)	Myzomyia
*Cellia*	*mascarensis* de Meillon, 1947	Neomyzomyia
*Cellia*	*melas* (Theobald, 1903)	Pyretophorus
*Cellia*	*merus* Dönitz, 1902	Pyretophorus
*Cellia*	*millecampsi* Lips, 1960	Neomyzomyia
*Cellia*	*milloti* Grjebine & Lacan, 1953	Neomyzomyia
*Cellia*	*mortiauxi* Edwards, 1938	Myzomyia
*Cellia*	*moucheti* Evans, 1925	Myzomyia
*Cellia*	*mousinhoi* de Meillon & de Carvalho Pereira, 1940	Myzomyia
*Cellia*	*multicolor* Cambouliu, 1902	Paramyzomyia
*Cellia*	*multicinctus* Edwards, 1930	Neomyzomyia
*Cellia*	*murphyi* Gillies & de Meillon, 1968	Cellia
*Cellia*	*natalensis* (Hill & Haydon, 1907)	Neomyzomyia
*Cellia*	*nili* (Theobald, 1904)	Neomyzomyia
*Cellia*	*njombiensis* Peters, 1955	Myzomyia
*Cellia*	*notleyi* van Someren, 1949	Neomyzomyia
*Cellia*	*ovengensis* Awono-Ambene, Kengne, Simard, Antonio-Nkondjio & Fontenille, 2004	Neomyzomyia
*Cellia*	*parensis* Gillies, 1962	Myzomyia
*Cellia*	*pauliani* Grjebine, 1953	Neomyzomyia
*Cellia*	*pharoensis* Theobald, 1901	Cellia
*Cellia*	*pretoriensis* (Theobald, 1903)	Neocellia
*Cellia*	*quadriannulatus* (Theobald, 1911)	Pyretophorus
*Cellia*	*radama* de Meillon, 1943	Neomyzomyia
*Cellia*	*rageaui* Mattingly & Adam, 1954	Neomyzomyia
*Cellia*	*ranci* Grjebine, 1953	Neomyzomyia
*Cellia*	*rhodesiensis* Theobald, 1901	Neomyzomyia
*Cellia*	*rivulorum* Leeson, 1935	Myzomyia
*Cellia*	*rivulorum-*like species (see Cohuet *et al*. 2003)	Myzomyia
*Cellia*	*rodhaini* Leleup & Lips, 1950	Neomyzomyia
*Cellia*	*roubaudi* Grjebine, 1953	Neomyzomyia
*Cellia*	*ruarinus* Edwards, 1940	Neomyzomyia
*Cellia*	*rufipes* (Gough, 1910)	Neocellia
*Cellia*	*salbaii* Maffi & Coluzzi, 1958	Neocellia
*Cellia*	*schwetzi* Evans, 1934	Myzomyia
*Cellia*	*seretsei* Abdulla-Khan, Coetzee & Hunt, 1998	Paramyzomyia
*Cellia*	*sergentii* (Theobald, 1907)	Myzomyia
*Cellia*	*seydeli* Edwards, 1929	Myzomyia
*Cellia*	*smithii* Theobald, 1905	Neomyzomyia
*Cellia*	*somalicus* Rivola & Holstein, 1957	Neomyzomyia
*Cellia*	*squamosus* Theobald, 1901	Cellia
*Cellia*	*stephensi* Liston, 1901	Neocellia
*Cellia*	*swahilicus* Gillies, 1964	Cellia
*Cellia*	*tchekedii* de Meillon & Leeson, 1940	Myzomyia
*Cellia*	*theileri* Edwards, 1912	Myzomyia
*Cellia*	*turkhudi* Liston, 1901	Paramyzomyia
*Cellia*	*vaneedeni* Gillies & Coetzee, 1987	Myzomyia
*Cellia*	*vanhoofi* Wanson & Lebied, 1945	Neomyzomyia
*Cellia*	*vernus* Gillies & de Meillon, 1968	Neomyzomyia
*Cellia*	*vinckei* de Meillon, 1942	Neomyzomyia
*Cellia*	*walravensi* Edwards, 1930	Myzomyia
*Cellia*	*wellcomei* Theobald, 1904	Myzomyia
*Cellia*	*wilsoni* Evans, 1934	Neomyzomyia
Christya*	*implexus* (Theobald, 1903)	–
*Christya*	*okuensis* Brunhes, Le Goff & Geoffroy, 1997	–

* Elevated to subgeneric level by Harbach & Kitching (2016).
