# Supplementary material for: Updated list of Anopheles species (Diptera: Culicidae) by country in the Afrotropical Region and associated islands
Source: Zootaxa. Author manuscript; Available in PMC 2020 Nov 5. (PMC7116328; doi:10.11646/zootaxa.4747.3.1)
Supplement: Appendix [file EMS102269-supplement-Appendix.pdf]

APPENDIX. Lists of *Anopheles* species recorded from Afrotropical countries, with notes on changes from the lists presented in Kyalo *et al*. (2017).

Angola

1) *An. arabiensis*
2) *An. ardensis*
3) *An. argenteolobatus*
4) *An. austenii*
5) *An. azevedoi*
6) *An. barberellus*
7) *An. brunnipes*
8) *An. caliginosus*
9) *An. cinctus*
10) *An. coluzzii*
11) *An. concolor*
12) *An. coustani*
13) *An. cydippis*
14) *An. demeilloni*
15) *An. distinctus*

17) *An. flavicosta*
18) *An. funestus*
19) *An. fuscivenosus*
20) *An. gambiae s.s.*
21) *An. hancocki*
22) *An. harperi*
23) *An. implexus*
24) *An. jebudensis*
25) *An. leesoni*
26) *An. listeri*
27) *An. longipalpis*
28) *An. maculipalpis*
29) *An. marshallii*
30) *An. melas*
31) *An. natalensis*
32) *An. nili*
33) *An. njombiensis*
34) *An. obscurus*
35) *An. paludis*
36) *An. pharoensis*
37) *An. pretoriensis*
38) *An. rhodesiensis*
39) *An. rivulorum*
40) *An. ruarinus*
41) *An. rufipes*
42) *An. schwetzi*
43) *An. squamosus*
44) *An. tchekedii*
45) *An. tenebrosus*
46) *An. theileri*
47) *An. walravensi*
48) *An. wellcomei*
49) *An. ziemanni*

Comments

1) Kyalo *et al.* (2017) list *An. walravensi*, which is not in the Africa Vectors database (Snow 2017), but was recorded from Angola by Gandara (1956) and is given in Gillies & de Meillon (1968).

2) The WRBU list did not have *An. jebudensis*, although this species is noted in de Barros Machado *et al.* (1981) and Gillies & Coetzee (1987).

3) The WRBU list, Gillies & de Meillon (1968), Gillies & Coetzee (1987) and the Kyalo *et al.* (2017) database do not mention *An. hancocki*. However, a specimen from Dundo, Angola was found in the Natural History Museum, London, so it is included here.

Benin

1) *An. arabiensis*
2) *An. brohieri*
3) *An. brunnipes*
4) *An. coluzzii*
5) *An. coustani*
6) *An. domicolus*
7) *An. flavicosta*

8) *An. funestus*
9) *An. gambiae s.s.*
10) *An. hargreavesi*
11) *An. leesoni*
12) *An. maculipalpis*
13) *An. melas*
14) *An. nili*
15) *An. obscurus*
16) *An. paludis*
17) *An. pharoensis*
18) *An. pretoriensis*
19) *An. rhodesiensis*
20) *An. rivulorum*
21) *An. rufipes*
22) *An. squamosus*
23) *An. wellcomei*
24) *An. ziemanni*

Comments

1) Kyalo *et al.* (2017) list *An. fuscicolor*, which is not in the Africa Vectors database (Snow 2017) (type locality in Madagascar, and the WRBU lists its distribution as only Madagascar). This record is from Gnanguenon *et al.* (2014), who mention the presence of *An. fuscicolor* in Benin. However, it is not a species found in the keys that Gnanguenon *et al.* used for identification (Gillies & de Meillon 1968; Gillies & Coetzee 1987), so we assume that the record in Gnanguenon is a misspelling of *An. fuscivenosus*, which is morphologically very similar to *An. funestus* but whose presence in Benin will need to be confirmed. We have therefore not included *An. fuscicolor* here.

2) Kyalo *et al.* (2017) also list *An. smithii* (the WRBU lists *An. smithii* as present in Nigeria and Ghana, so its presence in Benin would not be unexpected) but it is not in the Africa Vectors database (Snow 2017). The "Togo and Dahomey" country list of Gillies & de Meillon (1968) includes this species but it is not clear to which country it is referring; hence, the species is not included in the list here pending confirmation of this species in Benin.

Botswana

1) *An. arabiensis*
2) *An. argenteolobatus*
3) *An. caliginosus*
4) *An. coustani*
5) *An. cydippis*
6) *An. demeilloni*
7) *An. distinctus*
8) *An. funestus*
9) *An. leesoni*
10) *An. listeri*
11) *An. longipalpis*
12) *An. maculipalpis*
13) *An. marshallii*
14) *An. nili*
15) *An. parensis*
16) *An. pharoensis*
17) *An. pretoriensis*
18) *An. quadriannulatus*
19) *An. rhodesiensis*
20) *An. rivulorum*

21) *An. rufipes*
22) *An. seretsei*
23) *An. squamosus*
24) *An. tchekedii*
25) *An. tenebrosus*
26) *An. vaneedeni*
27) *An. walravensi*
28) *An. wellcomei*
29) *An. ziemanni*

Comments

1) Kyalo *et al.* (2017) list *An. cydippis*, *An. walravensi*, *An. wellcomei ugandae* and *An. ziemanni*, which were not in the Africa Vectors database (Snow 2017). Gillies & de Meillon (1968) list all of these as present in Botswana. Chayabejara *et al.* (1975) records *An. wellcomei ugandae*, *An. ziemanni* and *An. caliginosus.* The World Health Organization (1963) records *An. argenteolobatus*.

2) The WRBU lists *An. nili* as being present in Botswana, as does Gillies & de Meillon (1968) (in the text and distribution map (fig. 24), but not in the country list). We include it here.

3) *Anopheles funestus* was recorded from northwest Botswana by Koekemoer *et al.* (1999).

4) No records of *An. gambiae s.s.* have been found.

Burkina Faso

1) *An. arabiensis*
2) *An. argenteolobatus*
3) *An. brohieri*
4) *An. brumpti*
5) *An. brunnipes*
6) *An. coluzzii*
7) *An. coustani*
8) *An. cydippis*
9) *An. domicolus*
10) *An. dureni*
11) *An. flavicosta*
12) *An. freetownensis*
13) *An. funestus*
14) *An. gambiae s.s.*
15) *An. hancocki*
16) *An. implexus*
17) *An. leesoni*
18) *An. longipalpis*
19) *An. maculipalpis*
20) *An. murphyi*
21) *An. natalensis*
22) *An. nili*
23) *An. obscurus*
24) *An. paludis*
25) *An. pharoensis*
26) *An. pretoriensis*
27) *An. rhodesiensis*
28) *An. rivulorum*
29) *An. rufipes*
30) *An. sergentii*

31) *An. somalicus*
32) *An. squamosus*
33) *An. theileri*
34) *An. wellcomei*
35) *An. ziemanni*

Comments

1) Kyalo *et al.* (2017) list *An. subpictus* Grassi, which is primarily found in Australia and Asia. As it seems unlikely to be present in Burkina Faso (also no records were found in the database), it is not listed here. The Table also lists *An. sergentii*—*Anopheles macmahoni* (a subspecies of *An. sergentii*) was recorded by Choumara *et al.* (1959).

2) Choumara *et al.* (1959) also listed *An. paludis*, *An. rhodesiensis*, *An. obscurus*, *An. implexus* and *An. freetownensis* as being present in the pilot area, so they are included here.

3) Hamon *et al.* (1966) listed a number of other species as being present in Burkina Faso, including *An. paludis, An. theileri*, *An. obscurus*, *An. implexus*, *An. brunnipes*, *An. somalicus, An. cydippis*, *An. rhodesiensis* and *An. freetownensis*. *Anopheles brumpti* is shown as being on the border of Mali and Burkina Faso, and this is probably a record from Hamon & Rickenbach (1955), the collection which resulted in the description of *An. brumpti*.

4) Gillies & de Meillon (1968) listed *An. murphyi* for Burkina Faso.

5) Anopheles moucheti was mentioned by Robert (1989) in his thesis, but he does not cite any records from Burkina Faso, so this species is not included on the list.

Burundi

1) *An. arabiensis*
2) *An. brohieri*
3) *An. christyi*
4) *An. coustani*
5) *An. cydippis*
6) *An. demeilloni*
7) *An. funestus*
8) *An. gambiae s.l.*
9) *An. garnhami*
10) *An. gibbinsi*
11) *An. implexus*
12) *An. longipalpis*
13) *An. maculipalpis*
14) *An. marshallii*
15) *An. moucheti*
16) *An. natalensis*
17) *An. nili*
18) *An. pharoensis*
19) *An. seydeli*
20) *An. squamosus*
21) *An. theileri*
22) *An. ziemanni*

Comments

1) Kyalo *et al.* (2017) list *An. ardensis* and *An. wellcomei ugandae* as being present in Burundi but they are not in the database. A record for *An. ardensis* was found in Vincke & Jadin (1946), which describes it in nearby Democratic Republic of the Congo. Both *An. ardensis* and *An. wellcomei* are listed in Gillies & de Meillon (1968) but as these authors combined Rwanda and Burundi in their country lists, the presence of these species in Burundi is not confirmed, and they are not included in the present list pending future confirmation.

2) The Africa Indoor Residual Spray program included *An. brohieri* in their 2014 report (Africa Indoor Residual Spraying

(AIRS) Project 2014).

3) Anopheles gambiae s.l. appears not to have been tested for the molecular forms M and S or the chromosomal forms Mopti and Savanna.

Cabo Verde

1) *An. arabiensis*
2) *An. pretoriensis*

Comment

1) Ribeiro *et al.* (1979) described *An. quadriannulatus davidsoni* as a new sub-species from Cape Verde, but this was subsequently shown to be *An. arabiensis* by Cambournac *et al.* (1982) and "*davidsoni*" was synonymized with it.

Cameroon

1) *An. arabiensis*
2) *An. bervoetsi*
3) *An. brohieri*
4) *An. brunnipes*
5) *An. buxtoni*
6) *An. carnevalei*
7) *An. christyi*
8) *An. cinctus*
9) *An. coluzzii*
10) *An. concolor*
11) *An. coustani*
12) *An. cydippis*
13) *An. deemingi*
14) *An. demeilloni*
15) *An. domicolus*
16) *An. dualaensis*
17) *An. eouzani*
18) *An. flavicosta*
19) *An. freetownensis*
20) *An. funestus*
21) *An. gambiae s.s.*
22) *An. hancocki*
23) *An. hargreavesi*
24) *An. implexus*
25) *An. jebudensis*
26) *An. leesoni*
27) *An. longipalpis*
28) *An. maculipalpis*
29) *An. marshallii*
30) *An. melas*
31) *An. moucheti*
32) *An. mousinhoi*
33) *An. multicinctus*
34) *An. namibiensis*
35) *An. natalensis*
36) *An. nili*
37) *An. obscurus*

38) *An. okuensis*
39) *An. ovengensis.*
40) *An. paludis*
41) *An. pharoensis*
42) *An. pretoriensis*
43) *An. rageaui*
44) *An. rhodesiensis*
45) *An. rivulorum*
46) *An. rivulorum*-like
47) *An. rufipes*
48) *An. sergentii*
49) *An. smithii*
50) *An. somalicus*
51) *An. squamosus*
52) *An. tenebrosus*
53) *An. wellcomei*
54) *An. ziemanni*

Comments

1) Kyalo *et al.* (2017) list *An. deemingi* (as *An. deeming*), *An. eouzani* and *An. multicinctus* with no references in the database. Brunhes *et al.* (2003) described *An. deemingi* and *An. eouzani* from western Cameroon. Mouchet *et al.* (1960) described *An. multicinctus.*

2) As *An. nigeriensis* refers to a subspecies of *An. moucheti*, it is not included here.

3) Brunhes *et al*. (1997) described *An. okuensis* and Awono-Ambene *et al*. (2004) described *An. ovengensis* from Cameroon.

4) The only record found for *An. theileri* is in Adam & Mouchet (1957), who examined a specimen in a poor state, which seemed to be *An. theileri*. They stated that this finding should not be considered a record of collection, but should spur further research to see if the species is present in Cameroon. It has been removed from the list.

5) A new species not yet formally named, i.e. *An. rivulorum*-like (see Cohuet *et al.* 2003), is included in the list.

6) In the IRD database, *An. vaneedeni* was noted from Cameroon. As *An. vaneedeni* has only previously been found in eastern and southern Africa, this species is not added to the Cameroon list pending confirmation.

Central African Republic

1) *An. bervoetsi*
2) *An. brohieri*
3) *An. cinctus*
4) *An. coluzzii*
5) *An. coustani*
6) *An. cydippis*
7) *An. domicolus*
8) *An. flavicosta*
9) *An. freetownensis*
10) *An. funestus*
11) *An. gambiae s.s.*
12) *An. gibbinsi*
13) *An. hancocki*
14) *An. hargreavesi*
15) *An. implexus*
16) *An. leesoni*
17) *An. maculipalpis*
18) *An. marshallii*
19) *An. moucheti*

20) *An. multicinctus*
21) *An. natalensis*
22) *An. nili*
23) *An. obscurus*
24) *An. paludis*
25) *An. pharoensis*
26) *An. pretoriensis*
27) *An. rhodesiensis*
28) *An. rufipes*
29) *An. squamosus*
30) *An. wellcomei*
31) *An. ziemanni*

Comments

1) Geoffroy (1982) recorded *An. moucheti gibbinsi* and *An. natalensis multicinctus*, both of which have been elevated to species status, the former by Gillies & de Meillon (1968) and the latter by Brunhes *et al.* (1998).

2) Anopheles longipalpis is included in the list of Kyalo *et al.* (2017), but the two supporting references do not indicate a clear collection record. Rickenbach (1969) only referred to *Aedes longipalpis* (not *An. longipalpis*) and Hamon *et al.* (1956) do not distinguish between *An. domicolus* and *An. longipalpis* in reporting these species from Oubangui-Chari, so it is not clear that both are present in the country. For this reason, *An. longipalpis* has been removed from list.

Chad

1) *An. arabiensis*
2) *An. christyi*
3) *An. cinereus*
4) *An. coluzzii*
5) *An. coustani*
6) *An. cydippis*
7) *An. dthali*
8) *An. funestus*
9) *An. gambiae s.s.*
10) *An. nili*
11) *An. pharoensis*
12) *An. rhodesiensis*
13) *An. rufipes*
14) *An. sergentii*
15) *An. squamosus*
16) *An. tenebrosus*
17) *An. wellcomei*
18) *An. ziemanni*

Comments

1) Kyalo *et al.* (2017) list *An. rhodesiensis* and *An. dthali* based on the records provided by Rioux (1961), who discussed northern Chad (north of the 17th parallel used to define sub-Saharan Africa). Both species are recorded in Gillies & de Meillon (1968) and therefore remain on the list.

2) Regarding *An. cinereus*, it is not clear whether the mosquitoes collected by Rioux (1961) were *An. hispaniola* or *An. cinereus*, and according to Gillies & de Meillon (1968) the occurrence of *An. cinereus* this far west has not been confirmed. *Anopheles cinereus* remains on the list with Rioux (1961) as the justification, but we acknowledge that its presence requires confirmation.

3) Kyalo *et al.* (2017) list *An. cinctus* but this seems to be an error, as this species is not listed for Chad by Lacan (1958) or Gillies & de Meillon (1968). It is removed from the list.

4) Buck *et al.* (1968) found *An. coustani* var. *tenebrosus*, which has since been elevated to species rank.

Comoros

1) *An. comorensis*
2) *An. coustani*
3) *An. funestus*
4) *An. gambiae s.s.*
5) *An. maculipalpis*
6) *An. mascarensis*
7) *An. merus*
8) *An. pretoriensis*

Comment

1) Kyalo *et al*. (2017) list *An. arabiensis* and in the database referred to Brunhes (1977), but *An. arabiensis* is not referred to in this document. Similarly, no reference to *An. coluzzii* was found, so this is also removed from the list.

Côte d'Ivoire

1) *An. barberellus*
2) *An. brohieri*
3) *An. brunnipes*
4) *An. carnevalei*
5) *An. cinctus*
6) *An. coluzzii*
7) *An. coustani*
8) *An. demeilloni*
9) *An. domicolus*
10) *An. dureni*
11) *An. flavicosta*
12) *An. freetownensis*
13) *An. funestus*
14) *An. gambiae s.s.*
15) *An. hancocki*
16) *An. hargreavesi*
17) *An. implexus*
18) *An. jebudensis*
19) *An. leesoni*
20) *An. maculipalpis*
21) *An. marshallii*
22) *An. melas*
23) *An. moucheti*
24) *An. natalensis*
25) *An. nili*
26) *An. obscurus*
27) *An. paludis*
28) *An. pharoensis*
29) *An. pretoriensis*
30) *An. rageaui*
31) *An. rhodesiensis*
32) *An. rivulorum*
33) *An. rivulorum*-like
34) *An. rodhaini*
35) *An. rufipes*
36) *An. sergentii*

37) *An. smithii*
38) *An. somalicus*
39) *An. squamosus*
40) *An. wellcomei*
41) *An. ziemanni*

Comments

1) Kyalo *et al.* (2017) list *An. arabiensis*, but this species is not mentioned in the literature cited.
2) Kyalo *et al.* (2017) did not list *An. carnevalei* but this species is referenced in the database.
3) *Anopheles rageaui* was recorded as *An. smithii rageaui* by Adam & Hamon (1958). Hamon *et al.* (1962) report *An. smithii* in Man (western Côte d'Ivoire). Both species are included in the list.
4) *Anopheles rivulorum*-like was found in Ganse (Adja *et al.* 2006).
5) The record of *An. marshallii* in Doucet *et al.* (1960) was always preceded by a question mark. The species is included in the country lists of Gillies & de Meillon (1968), although they suggest that West African records of *An. marshallii* may have been confused with *An. hargreavesi*. *Anopheles marshallii* is retained on the list here with the caveat that more collections are needed to confirm its presence in Côte d'Ivoire.
6) The only record for *An. rodhaini* is that of Adam (1965), who considered it to be a dark form of *An. rhodesiensis*. It seems Adam was using this example to show how close *An. rodhaini* is to *An. rhodesiensis*, rather than recording *An. rodhaini* in Côte d'Ivoire. It has been removed from the list.
7) In the IRD database, *An. longipalpis* was noted from Cameroon. As *An. longipalpis* has only previously been found in eastern and southern Africa, this species is not added to the Côte d'Ivoire list pending confirmation.

Democratic Republic of Congo

1) *An. arabiensis*
2) *An. ardensis*
3) *An. argenteolobatus*
4) *An. austenii*
5) *An. barberellus*
6) *An. berghei*
7) *An. bervoetsi*
8) *An. brunnipes*
9) *An. caliginosus*
10) *An. christyi*
11) *An. cinctus*
12) *An. coluzzii*
13) *An. concolor*
14) *An. confusus*
15) *An. coustani*
16) *An. cydippis*
17) *An. demeilloni*
18) *An. distinctus*
19) *An. domicolus*
20) *An. dureni*
21) *An. faini*
22) *An. funestus*
23) *An. gambiae s.s.*
24) *An. garnhami*
25) *An. gibbinsi*
26) *An. hancocki*
27) *An. hargreavesi*
28) *An. implexus*
29) *An. jebudensis*

30) *An. keniensis*
31) *An. kingi*
32) *An. leesoni*
33) *An. longipalpis*
34) *An. maculipalpis*
35) *An. marshallii*
36) *An. melas*
37) *An. millecampsi*
38) *An. mortiauxi*
39) *An. moucheti*
40) *An. mousinhoi*
41) *An. multicinctus*
42) *An. natalensis*
43) *An. nili*
44) *An. njombiensis*
45) *An. obscurus*
46) *An. paludis*
47) *An. pharoensis*
48) *An. pretoriensis*
49) *An. rageaui*
50) *An. rhodesiensis*
51) *An. rivulorum*
52) *An. rodhaini*
53) *An. rufipes*
54) *An. schwetzi*
55) *An. seydeli*
56) *An. squamosus*
57) *An. symesi*
58) *An. tenebrosus*
59) *An. theileri*
60) *An. vanhoofi*
61) *An. vinckei*
62) *An. walravensi*
63) *An. wellcomei*
64) *An. ziemanni*

Comments

1) Kyalo *et al.* (2017) list *An. jebudensis* and *An. njombiensis*, which are not in the database. *Anopheles jebudensis* was collected by Vincke & Laarman (1956) in Kivu. *Anopheles njombiensis* is listed in Gillies & de Meillon (1968).
2) The mention of *An. ugandae* may refer to *An. distinctus ugandae* listed in Rahm & Vermylen (1967) or to the subspecies of *An. wellcomei* listed in Gillies & de Meillon (1968). In either case it is at most a subspecies and is removed from the list.
3) *Anopheles cinctus* is listed by Kyalo *et al.* (2017) based on Rahm & Vermylen (1967), but there is no mention of *An. cinctus* by those authors. However, Vincke & Laarman (1956) record this species.
4) *Anopheles millecampsi* was collected from Lubumbashi by Vincke (1946), although it was not clear in that publication that he had collected a new species. It was formally recognized as a species by Brunhes *et al.* (1997).

Djibouti

1) *An. arabiensis*
2) *An. azaniae*
3) *An. dancalicus*
4) *An. dthali*
5) *An. harperi*
6) *An. pharoensis*

7) *An. rhodesiensis*
8) *An. salbaii*
9) *An. sergentii*
10) *An. stephensi*
11) *An. turkhudi*

Comments

1) Kyalo *et al.* (2017) list *An. gambiae s.l.*, and although no references for *An. arabiensis* have been found, the World Health Organization (1990, 2014) recognize it as a principle vector of malaria in Djibouti.
2) Carteron *et al.* (1978) noted the presence of *An. pharoensis*.
3) Faulde *et al.* (2014) and Seyfarth *et al.* (2019) reported the presence of *An. stephensi* in Djibouti. This is an introduced species, found in the Middle East and southern Asian countries.

Equatorial Guinea

1) *An. brunnipes*
2) *An. carnevalei*
3) *An. cinctus*
4) *An. coluzzii*
5) *An. funestus*
6) *An. gambiae s.s.*
7) *An. leesoni*
8) *An. lloreti*
9) *An. melas*
10) *An. moucheti*
11) *An. obscurus*
12) *An. ovengensis*
13) *An. smithii*

Comments

1) While Kyalo *et al.* (2017) mention *An. ovengensis* in their database (and it is in Ridl *et al.* 2008), this species was not included in their list. It is included in the list here.
2) Pappa *et al.* (2011) recorded *An. leesoni*, which has been added to the list.
3) No records were found for *An. nili* (surprisingly). Only records for *An. carnevalei* and *An. ovengensis* of the *An. nili* complex were found. *Anopheles nili* is removed from the list.

Eritrea

1) *An. arabiensis*
2) *An. christyi*
3) *An. cinereus*
4) *An. coustani*
5) *An. culicifacies*
6) *An. dancalicus*
7) *An. demeilloni*
8) *An. dthali*
9) *An. erythraeus*
10) *An. funestus*
11) *An. garnhami*
12) *An. harperi*
13) *An. pharoensis*
14) *An. pretoriensis*

15) *An. rhodesiensis*
16) *An. rivulorum*
17) *An. rufipes*
18) *An. salbaii*
19) *An. sergentii*
20) *An. squamosus*
21) *An. turkhudi*
22) *An. wellcomei*

Comments

1) Kyalo *et al.* (2017) list *An. rhodesiensis rupicolus*. As this is a subspecies of *An. rhodesiensis*, it has been removed as a separate entity.
2) Kyalo *et al.* (2017) do not list *An. pretoriensis*, but this species was recorded by Raffaele (1942).
3) Anopheles culicifacies was recorded by Jannone *et al.* (1946) and is added to the list.
4) Shililu *et al.* (2003) recorded *An. wellcomei* and *An. harperi*.
5) The only record for *An. nili* seems to come from Nyanjom *et al.* (2003), who do not indicate where the *An. nili* were collected (Ethiopia or Eritrea). *Anopheles nili* has been removed from the list.
6) A specimen of *Anopheles salbaii* from Arafali is in the collection of the Natural History Museum, London.

Eswatini (previously Swaziland)

1) *An. arabiensis*
2) *An. caliginosus*
3) *An. cinereus*
4) *An. coustani*
5) *An. cydippis*
6) *An. funestus*
7) *An. gambiae s.s.*
8) *An. maculipalpis*
9) *An. marshallii*
10) *An. merus*
11) *An. nili*
12) *An. pretoriensis*
13) *An. quadriannulatus*
14) *An. rhodesiensis*
15) *An. rivulorum*
16) *An. ruarinus*
17) *An. rufipes*
18) *An. squamosus*

Comments

1) Kyalo *et al.* (2017) do not list *An. gambiae s.s.* However, de Almeida Franco *et al.* (1984), page 40, mentioned that G.E. Bakri, a WHO entomologist who visited Swaziland in 1978, recorded the collection of 95 *An. gambiae* (species A), 80 *An. arabiensis*, seven *An. quadriannulatus* and one *An. merus*. All were identified cytogenetically by Davidson and Coluzzi. *Anopheles gambiae s.s.* has been added to the list.
2) Anopheles cinereus, *An. cydippis*, *An. rhodesiensis* and *An. ruarinus* were found in the National Institute for Communicable Diseases/National Health Laboratory Service collection (Johannesburg, South Africa).

Ethiopia

1) *An. amharicus*
2) *An. arabiensis*
3) *An. ardensis*

4) *An. azaniae*
5) *An. christyi*
6) *An. cinctus*
7) *An. cinereus*
8) *An. confusus*
9) *An. coustani*
10) *An. culicifacies*
11) *An. cydippis*
12) *An. dancalicus*
13) *An. demeilloni*
14) *An. domicolus*
15) *An. dthali*
16) *An. ethiopicus*
17) *An. funestus*
18) *An. garnhami*
19) *An. gibbinsi*
20) *An. harperi*
21) *An. implexus*
22) *An. kingi*
23) *An. leesoni*
24) *An. longipalpis*
25) *An. maculipalpis*
26) *An. marshallii*
27) *An. natalensis*
28) *An. nili*
29) *An. obscurus*
30) *An. paludis*
31) *An. parensis*
32) *An. pharoensis*
33) *An. pretoriensis*
34) *An. rhodesiensis*
35) *An. rivulorum*
36) *An. rufipes*
37) *An. salbaii*
38) *An. sergentii*
39) *An. seydeli*
40) *An. squamosus*
41) *An. stephensi*
42) *An. tenebrosus*
43) *An. theileri*
44) *An. turkhudi*
45) *An. wellcomei*
46) *An. ziemanni*

Comments

1) Kyalo *et al.* (2017) list *An. erythraeus*, which is mentioned by Giaquinto-Mira (1950), but this is a record in Eritrea only, not in present day Ethiopia and O'Connor (1967) removed the species from his list.

2) Kyalo *et al.* (2017) do not list *An. cinctus* but this species is reported by Massebo & Lindtjørn (2013).

3) Gillies & de Meillon (1968) noted that *An. theileri* listed by Verrone (1962a, b) possibly refers to *An. brohieri* (adult) or *An. wellcomei* (larva), but as this species is reported by O'Connor (1967) it remains on the list.

4) Carter *et al.* (2018) record the presence of *An. stephensi* in Ethiopia. This is an introduced species, found in the Middle East and southern Asian countries.

5) A specimen of *An. ethiopicus* collected in Gambella is deposited in the collection in the Natural History Museum, London.
6) *Anopheles azaniae* was collected by Animut *et al.* (2012).

Gabon

1) *An. carnevalei*
2) *An. caroni*
3) *An. cinctus*
4) *An. coluzzii*
5) *An. coustani*
6) *An. eouzani*
7) *An. faini*
8) *An. fontenillei*
9) *An. funestus*
10) *An. gabonensis*
11) *An. gambiae s.s.*
12) *An. hancocki*
13) *An. hargreavesi*
14) *An. implexus*
15) *An. jebudensis*
16) *An. maculipalpis*
17) *An. marshallii*
18) *An. melas*
19) *An. moucheti*
20) *An. natalensis*
21) *An. nili*
22) *An. obscurus*
23) *An. paludis*
24) *An. pharoensis*
25) *An. pretoriensis*
26) *An. rhodesiensis*
27) *An. rufipes*
28) *An. schwetzi*
29) *An. smithii*
30) *An. squamosus*
31) *An. tenebrosus*
32) *An. theileri*
33) *An. vinckei*
34) *An. wellcomei*
35) *An. ziemanni*

Comments

1) Kyalo *et al.* (2017) do not include *An. carnevalei* or *An. eouzani*, which were found by Paupy *et al.* (2013) and have been added to the list.
2) Elissa *et al.* (1999) reported *An. squamosus*, which has been added to the list.
3) Barrón *et al*. (2019) described *An. fontenillei* from the National Park of La Lopé in central Gabon, based primarily on molecular and cytogenic analyses.

The Gambia

1) *An. arabiensis*

2) *An. brohieri*
3) *An. brucei*
4) *An. brunnipes*
5) *An. coluzzii*
6) *An. coustani*
7) *An. flavicosta*
8) *An. funestus*
9) *An. gambiae s.s.*
10) *An. maculipalpis*
11) *An. melas*
12) *An. murphyi*
13) *An. nili*
14) *An. pharoensis*
15) *An. rufipes*
16) *An. squamosus*
17) *An. wellcomei*
18) *An. ziemanni*

Comment

1) There is a specimen of *An. brucei* from Tambana in the Natural History Museum, London. On the label, the identification is written as "*A. brucei*?"

Ghana

1) *An. arabiensis*
2) *An. brohieri*
3) *An. brunnipes*
4) *An. cinctus*
5) *An. coluzzii*
6) *An. coustani*
7) *An. demeilloni*
8) *An. domicolus*
9) *An. flavicosta*
10) *An. freetownensis*
11) *An. funestus*
12) *An. gambiae s.s.*
13) *An. hancocki*
14) *An. hargreavesi*
15) *An. implexus*
16) *An. leesoni*
17) *An. maculipalpis*
18) *An. marshallii*
19) *An. melas*
20) *An. moucheti*
21) *An. nili*
22) *An. obscurus*
23) *An. paludis*
24) *An. pharoensis*
25) *An. pretoriensis*
26) *An. rageaui*
27) *An. rhodesiensis*
28) *An. rufipes*

29) *An. squamosus*
30) *An. wellcomei*
31) *An. ziemanni*

Comments

1) Kyalo *et al.* (2017) list *An. watsoni*, which is a synonym of *An. rufipes* and is not included in the list.
2) No reference was found for *An. theileri* that did not refer to *An. brohieri*, so it is not included on the list.
3) The Gillies & de Meillon (1968) queried the presence of *An. demeilloni*. This species is reported by Chinery (1995).
4) Brady (1965) reported *An. smithii* var. *rageaui.* This appears to be the only record of *An. smithii* in Ghana, and since it refers to *An. rageaui* (Brunhes *et al.* 1999), *An. rageaui* is added to the list and *An. smithii* is removed.
5) A specimen of *An. moucheti* is in the Natural History Museum, London.

Guinea

1) *An. arabiensis*
2) *An. barberellus*
3) *An. brohieri*
4) *An. brunnipes*
5) *An. cinctus*
6) *An. coluzzii*
7) *An. coustani*
8) *An. domicolus*
9) *An. flavicosta*
10) *An. freetownensis*
11) *An. funestus*
12) *An. gambiae s.s.*
13) *An. hancocki*
14) *An. hargreavesi*
15) *An. implexus*
16) *An. leesoni*
17) *An. longipalpis*
18) *An. maculipalpis*
19) *An. maliensis*
20) *An. marshallii*
21) *An. melas*
22) *An. moucheti*
23) *An. nili*
24) *An. obscurus*
25) *An. pharoensis*
26) *An. pretoriensis*
27) *An. rageaui*
28) *An. rhodesiensis*
29) *An. rivulorum*
30) *An. rufipes*
31) *An. sergentii*
32) *An. smithii*
33) *An. somalicus*
34) *An. squamosus*
35) *An. wellcomei*
36) *An. ziemanni*

Comment

1) Kyalo *et al.* (2017) list *An. cavernicolus*, *An. nigeriensis* and *An. sergentii macmahoni*. *Anopheles nigeriensis* and *An.*

sergentii macmahoni are represented on the species list as *An. moucheti* and *An. sergentii*, respectively. *Anopheles cavernicolus* was described from specimens collected near Dalaba, Guinea, (Abonnenc 1954) but was synonymized with *An. smithii* by Gillies & de Meillon (1968), and is deleted from the list.

Guinea-Bissau

1) *An. arabiensis*
2) *An. cinereus*
3) *An. coluzzii*
4) *An. coustani*
5) *An. funestus*
6) *An. gambiae s.s.*
7) *An. hargreavesi*
8) *An. maculipalpis*
9) *An. melas*
10) *An. nili*
11) *An. pharoensis*
12) *An. rufipes*
13) *An. smithii*
14) *An. squamosus*
15) *An. ziemanni*

Comment

1) Kyalo *et al.* (2017) list *An. dancalicus*, which was reported by Ferreira *et al.* (1948) but this record is so far from the known range of the species that it has been removed from the list. Furthermore, Gillies & de Meillon (1968) suggested this record was a misidentification of *An. maculipalpis*.

Kenya

1) *An. arabiensis*
2) *An. ardensis*
3) *An. azaniae*
4) *An. christyi*
5) *An. cinereus*
6) *An. confusus*
7) *An. coustani*
8) *An. demeilloni*
9) *An. dthali*
10) *An. erepens*
11) *An. flavicosta*
12) *An. funestus*
13) *An. gambiae s.s.*
14) *An. garnhami*
15) *An. gibbinsi*
16) *An. hancocki*
17) *An. harperi*
18) *An. implexus*
19) *An. keniensis*
20) *An. kingi*
21) *An. leesoni*
22) *An. longipalpis*
23) *An. lounibosi*

24) *An. maculipalpis*
25) *An. marshallii*
26) *An. merus*
27) *An. moucheti*
28) *An. multicinctus*
29) *An. natalensis*
30) *An. nili*
31) *An. paludis*
32) *An. parensis*
33) *An. pharoensis*
34) *An. pretoriensis*
35) *An. rhodesiensis*
36) *An. rivulorum*
37) *An. rufipes*
38) *An. salbaii*
39) *An. sergentii*
40) *An. smithii*
41) *An. squamosus*
42) *An. swahilicus*
43) *An. symesi*
44) *An. tenebrosus*
45) *An. theileri*
46) *An. vaneedeni*
47) *An. wellcomei*
48) *An. wilsoni*
49) *An. ziemanni*

Comments

1) Kyalo *et al.* (2017) list both *An. lounibosi* and *An. rabaiensis* Lounibos, as well as *An. salbaii* and *An. wellcomei erepens*. *Anopheles rabaiensis* is a *nomen nudum* for *An. lounibosi* (Gillies & Coetzee 1987) and is removed from the list. *Anopheles salbaii* is not in the Kyalo database but Cooke *et al.* (2015) reported this species so it is retained on the list. Gillies (1958) notes that *An. wellcomei erepens* has been found in the Taveta region of Kenya, and it has been included in the list as *An. wellcomei*.

2) Symes (1931) noted that *An. theileri* var. *hancocki* has been collected in several districts in Kenya. Evans (1938) noted that the description given by Symes (1931) is "evidently that of *hancocki*", which is included in the list.

3) A specimen of *An. erepens* from Taveta is in the Natural History Museum, London.

Lesotho

1) *An. carteri*

Comment

1) Two specimens of *An. carteri* were found in the National Institute for Communicable Diseases/National Health Laboratory Service collection (Johannesburg, South Africa). The specimens were initially identified as *An. demeilloni*, but were re-identified as *An. carteri* by M. Gillies, and confirmed by one of us (MC).

Liberia

1) *An. barberellus*
2) *An. cinctus*
3) *An. coluzzii*
4) *An. coustani*

5) *An. funestus*
6) *An. gambiae s.s.*
7) *An. hancocki*
8) *An. hargreavesi*
9) *An. melas*
10) *An. nili*
11) *An. obscurus*
12) *An. paludis*
13) *An. pretoriensis*
14) *An. smithii*
15) *An. squamosus*
16) *An. ziemanni*

Comments

1) Hamon *et al.* (1956) listed Liberia as one of the countries where *An. wellcomei* is present, but this is not included in the table in the same publication, and no collection site is provided in this document or a later paper on the distribution of *Anopheles* in West Africa (Hamon *et al.* 1966), so *An. wellcomei* is not included in the list. It is also not listed in Gillies & de Meillon (1968). However, Gelfand (1954) listed it as one of the mosquitoes he collected. Its presence in Liberia requires confirmation.

2) Records of *An. squamosus* are not provided in the references in the Kyalo *et al.* database. Gelfand (1954) includes *An. squamosus* in the identification key but does not say it was found in Liberia. Hamon *et al.* (1956) include it as present in Liberia, but do not provide collection data, and, similar to *An. wellcomei*, no records are provided in the later document (Hamon *et al.* 1966), but Gillies & de Meillon (1968) provided a location for the collection of *An. squamosus* in Liberia, so it is retained on the list. However, its presence in the country should be verified.

Madagascar

1) *An. arabiensis*
2) *An. brunnipes*
3) *An. coustani*
4) *An. cydippis*
5) *An. flavicosta*
6) *An. funestus*
7) *An. fuscicolor*
8) *An. gambiae s.s.*
9) *An. grassei*
10) *An. grenieri*
11) *An. griveaudi*
12) *An. lacani*
13) *An. maculipalpis*
14) *An. mascarensis*
15) *An. merus*
16) *An. milloti*
17) *An. notleyi*
18) *An. pauliani*
19) *An. pharoensis*
20) *An. pretoriensis*
21) *An. radama*
22) *An. ranci*
23) *An. roubaudi*
24) *An. rufipes*
25) *An. squamosus*
26) *An. tenebrosus*

Comment

1) Wilson (1947) recorded *An. marshallii* on Madagascar, however Chauvet (1962) determined, after examination of the cibarial armature, that all "*An. marshallii*" from Madagascar were actually *An. mascarensis*. *Anopheles marshallii* is also excluded from the extensive treatment of Madagascan *Anopheles* provided by Grjebine (1966). This species is therefore not included on the list.

2) The IRD database lists *An. nili* as being present in Madagascar, but due to the lack of any other records, this species has not been added to the list pending confirmation.

Malawi

1) *An. arabiensis*
2) *An. cinereus*
3) *An. coustani*
4) *An. demeilloni*
5) *An. distinctus*
6) *An. funestus*
7) *An. funestus*-like.
8) *An. gambiae s.s.*
9) *An. longipalpis*
10) *An. maculipalpis*
11) *An. marshallii*
12) *An. parensis*
13) *An. pharoensis*
14) *An. pretoriensis*
15) *An. quadriannulatus*
16) *An. rhodesiensis*
17) *An. rivulorum*
18) *An. rufipes*
19) *An. seydeli*
20) *An. squamosus*
21) *An. tenebrosus*
22) *An. ziemanni*

Comments

1) Mzilahowa *et al.* (2008) found *An. quadriannulatus* in the lower Shire Valley.
2) The as yet unnamed species, *An. funestus*-like, is included here (see Spillings *et al.* 2009).

Mali

1) *An. arabiensis*
2) *An. brohieri*
3) *An. brunnipes*
4) *An. coluzzii*
5) *An. coustani*
6) *An. domicolus*
7) *An. dthali*
8) *An. flavicosta*
9) *An. funestus*
10) *An. gambiae s.s.*
11) *An. hancocki*
12) *An. leesoni*
13) *An. maculipalpis*

14) *An. nili*
15) *An. obscurus*
16) *An. paludis*
17) *An. pharoensis*
18) *An. pretoriensis*
19) *An. rhodesiensis*
20) *An. rivulorum*
21) *An. rufipes*
22) *An. sergentii*
23) *An. squamosus*
24) *An. somalicus*
25) *An. wellcomei*
26) *An. ziemanni*

Comment

1) Kyalo *et al.* (2017) list *An. longipalpis*, which is recorded in Hamon *et al.* (1961), but this refers to *An. longipalpis domicolus* (*An. domicolus*), so it is removed from the list.

2) The IRD database includes *An. azaniae* and *An. turkhudi*. As these are far outside the normal distribution of the species, they are not included in the list pending confirmation.

Mauritania

1) *An. arabiensis*
2) *An. coluzzii*
3) *An. coustani*
4) *An. demeilloni*
5) *An. domicolus*
6) *An. dthali*
7) *An. freetownensis*
8) *An. funestus*
9) *An. melas*
10) *An. pharoensis*
11) *An. pretoriensis*
12) *An. rhodesiensis*
13) *An. rufipes*
14) *An. squamosus*
15) *An. wellcomei*
16) *An. ziemanni*

Comments

1) Kyalo *et al.* (2017) did not include *An. gambiae s.s.* because only *An. coluzzii* and *An. arabiensis* were identified when further analysis of *An. gambiae s.l.* mosquitoes was conducted.

2) *Anopheles wellcomei* was noted by Diallo *et al.* (2005) and is added to the list.

3) *Anopheles flavicosta* is stated to be present in Mauritania in the CD-ROM identification key by Hervy *et al.* (1998). As no other mention of this species can be found in the literature, it is left off the list.

4) Kyalo *et al.* (2017) included *An. hancocki*, with reference to Khromov (1969) in the database; however, this report contains no mention of *An. hancocki*, so it is removed from the list.

Mauritius

1) *An. arabiensis*
2) *An. coustani*
3) *An. maculipalpis*

4) *An. merus*

Comment

1) Anopheles gambiae s.s. (as *An. gambiae* species A) (Davidson 1966) and *An. funestus* of MacGregor (1924) were collected in Mauritius before the DDT and HCH (hexachlorocyclohexane) spraying campaigns. However, Bryan & Gerbert (1976) did not find *An. gambiae* species A, and Dowling (1953) reported an absence of *An. funestus*. Gopaul (1995) reported only four species of *Anopheles* in Mauritius, the same species listed above.

Mayotte

1) *An. coustani*
2) *An. funestus*
3) *An. gambiae s.s.*
4) *An. maculipalpis*
5) *An. mascarensis*
6) *An. merus*
7) *An. pretoriensis*

Comment

1) Grjebine (1966) noted the presence of *An. merus* on Mayotte, so it is added to the list.

Mozambique

1) *An. arabiensis*
2) *An. brunnipes*
3) *An. cinereus*
4) *An. confusus*
5) *An. coustani*
6) *An. cydippis*
7) *An. demeilloni*
8) *An. funestus*
9) *An. gambiae s.s.*
10) *An. leesoni*
11) *An. letabensis*
12) *An. listeri*
13) *An. longipalpis*
14) *An. maculipalpis*
15) *An. marshallii*
16) *An. merus*
17) *An. mousinhoi*
18) *An. natalensis*
19) *An. nili*
20) *An. parensis*
21) *An. pharoensis*
22) *An. pretoriensis*
23) *An. quadriannulatus*
24) *An. rhodesiensis*
25) *An. rivulorum*
26) *An. rufipes*
27) *An. seydeli*
28) *An. squamosus*
29) *An. tenebrosus*

30) *An. theileri*
31) *An. wellcomei*
32) *An. ziemanni*

Comments

1) Kyalo *et al.* (2017) included *An. paludis* as mentioned by Soromenho (1923). Soromenho's report is not accompanied by any description and probably refers to *An. coustani*, recorded extensively in surveys conducted in Mozambique by de Meillon & de Carvalho Pereira (1940). The distribution maps in Gillies & de Meillon (1968) show no records of *An. paludis* from Mozambique. It is therefore deleted from the list.
2) Gillies & de Meillon (1968) note that the presence of *An. domicolus* in Mozambique remains to be confirmed. It is therefore not included on the list here.
3) Cornel *et al.* (2018) reported *An. parensis* from Vilankulo, Inhambane Province in Mozambique, which is added to the list.

Namibia

1) *An. arabiensis*
2) *An. azevedoi*
3) *An. cinereus*
4) *An. coustani*
5) *An. demeilloni*
6) *An. distinctus*
7) *An. fontinalis*
8) *An. funestus*
9) *An. gambiae s.s.*
10) *An. listeri*
11) *An. maculipalpis*
12) *An. marshallii*
13) *An. namibiensis*
14) *An. nili*
15) *An. pharoensis*
16) *An. pretoriensis*
17) *An. quadriannulatus*
18) *An. rhodesiensis*
19) *An. rivulorum*
20) *An. ruarinus*
21) *An. rufipes*
22) *An. squamosus*
23) *An. vaneedeni*
24) *An. ziemanni*

Comments

1) Kyalo *et al.* (2017) list *An. moucheti*, found by de Meillon (1951) in the larval stage. Gillies & de Meillon (1968) noted that this record refers to either *An. domicolus* or *An. longipalpis*. Further confirmation of the species is needed and therefore none of the three species is included on the list here.
2) *Anopheles azevedoi* was collected as larvae in central Namibia (Ntomwa *et al.* 2006).

Niger

1) *An. arabiensis*
2) *An. cinereus*
3) *An. coluzzii*

4) *An. domicolus*
5) *An. dthali*
6) *An. flavicosta*
7) *An. funestus*
8) *An. gambiae s.s.*
9) *An. hervyi*
10) *An. maculipalpis*
11) *An. multicolor*
12) *An. nili*
13) *An. pharoensis*
14) *An. pretoriensis*
15) *An. rhodesiensis*
16) *An. rivulorum*
17) *An. rufipes*
18) *An. salbaii*
19) *An. squamosus*
20) *An. wellcomei*
21) *An. ziemanni*

Comments

1) Kyalo *et al.* (2017) included *An. rhodesiensis* and *An. rhodesiensis rupicolus*. The subspecies is removed from the list.
2) *Anopheles hervyi* was noted by Labbo *et al.* (2010) and is added to the list.
3) Julvez *et al.* (1998) lists *An. hispaniola*, *An. domicolus* and *An. flavicosta*, and these are added to the list. *Anopheles hispaniola* is added as *An. cinereus*, as it was synonymized with *An. cinereus* (Dahl & White 1978).
4) Julvez *et al.* (1998) stated that probably only *An. ziemanni* is present in Niger, and that records of *An. coustani* prior to 1970 did not distinguish between *An. coustani* and *An. ziemanni*. Until specific records of *An. coustani* are provided, this species is removed from the list.
5) The IRD database includes *An. moucheti*, but as this is far from its known distribution, it has not been included in the list pending confirmation.

Nigeria

1) *An. arabiensis*
2) *An. ardensis*
3) *An. barberellus*
4) *An. brohieri*
5) *An. brucei*
6) *An. brunnipes*
7) *An. cinctus*
8) *An. coluzzii*
9) *An. coustani*
10) *An. cristipalpis*
11) *An. deemingi*
12) *An. domicolus*
13) *An. flavicosta*
14) *An. freetownensis*
15) *An. funestus*
16) *An. gambiae s.s.*
17) *An. hancocki*
18) *An. hargreavesi*
19) *An. implexus*
20) *An. jebudensis*

21) *An. leesoni*
22) *An. maculipalpis*
23) *An. marshallii*
24) *An. melas*
25) *An. moucheti*
26) *An. nili*
27) *An. obscurus*
28) *An. paludis*
29) *An. pharoensis*
30) *An. pretoriensis*
31) *An. rhodesiensis*
32) *An. rivulorum*
33) *An. rufipes*
34) *An. smithii*
35) *An. squamosus*
36) *An. theileri*
37) *An. wellcomei*
38) *An. ziemanni*

Comments

1) Kyalo *et al.* (2017) list *An. watsoni* Edwards and *An. moucheti nigeriensis*. *Anopheles watsoni* is a synonym of *An. rufipes* and is not included in the list. *Anopheles moucheti nigeriensis* is a subspecies of *An. moucheti*, so is not included on the list.
2) Ayanda (2009) collected *An. ardensis*, so this is added to the list.
3) The type specimen of *An. deemingi* is in the Natural History Museum, London

Republic of Congo

1) *An. ardensis*
2) *An. barberellus*
3) *An. brohieri*
4) *An. brunnipes*
5) *An. caroni*
6) *An. cinctus*
7) *An. cinereus*
8) *An. coluzzii*
9) *An. coustani*
10) *An. cydippis*
11) *An. demeilloni*
12) *An. dureni*
13) *An. freetownensis*
14) *An. funestus*
15) *An. gambiae s.s.*
16) *An. gibbinsi*
17) *An. hamoni*
18) *An. hancocki*
19) *An. hargreavesi*
20) *An. implexus*
21) *An. jebudensis*
22) *An. leesoni*
23) *An. longipalpis*
24) *An. marshallii*
25) *An. melas*

26) *An. moucheti*
27) *An. natalensis*
28) *An. nili*
29) *An. obscurus*
30) *An. paludis*
31) *An. pretoriensis*
32) *An. rageaui*
33) *An. rhodesiensis*
34) *An. rivulorum*
35) *An. rufipes*
36) *An. squamosus*
37) *An. vanhoofi*
38) *An. ziemanni*

Comments

1) Adam (1964) indicated that larvae of *An. smithii rageaui* (now *An. rageaui*) had been found at Sounda by Taufflieb.
2) The record of *An. cinereus* seems out of the normal range of the species, but it is recorded by Lacan (1958) as being collected by Grjebine near Brazzaville. Lacan also recorded a collection of *An. gibbinsi* (as *An. moucheti gibbinsi*) in Moyen-Congo.
3) *Anopheles buxtoni* is included in the IRD database, but as this is far from its normal distribution, it is not included pending confirmation.

La Réunion

1) *An. arabiensis*
2) *An. coustani*

Comment

1) *Anopheles funestus* and *An. squamosus* have been recorded on La Réunion, but in light of the absence of any specimens in museums, Hamon & Dufour (1954) doubted the presence of these species on the island. In the absence of any recent collections or proof of previous collections, these species are not included on the list.

Rwanda

1) *An. arabiensis*
2) *An. christyi*
3) *An. coustani*
4) *An. cydippis*
5) *An. demeilloni*
6) *An. dureni*
7) *An. funestus*
8) *An. gambiae s.s.*
9) *An. garnhami*
10) *An. gibbinsi*
11) *An. implexus*
12) *An. maculipalpis*
13) *An. marshallii*
14) *An. moucheti*
15) *An. natalensis*
16) *An. paludis*
17) *An. pharoensis*
18) *An. pretoriensis*
19) *An. squamosus*

20) *An. tenebrosus*
21) *An. ziemanni*

Comments

1) Kyalo *et al.* (2017) did not include *An. cydippis*. Vincke & Jadin (1946) noted its presence in Astrida (Butare) as *An. squamosus entebbiensis*.

2) *Anopheles gibbinsi* is not listed by Kyalo *et al.* (2017) but was found by Vincke & Jadin (1946) and is added to the list.

3) We could not find records of *An. nili* or *An. ardensis* in Rwanda, however, they are listed as being present in Ruanda Urundi by Gillies & De Meillon (1968). Vermylen (1967) listed *An. nili* as being present in Burundi but not Rwanda. Until records of either species can be found, we remove these two species from the list.

São Tomé e Príncipe

1) *An. coluzzii*
2) *An. coustani*
3) *An. funestus*
4) *An. melas*
5) *An. paludis*
6) *An. pharoensis*

Comment

1) Kyalo *et al.* (2017) did not include *An. gambiae s.s*. Recent reports only indicate that *An. coluzzii* is present (della Torre *et al.* 2001; Marshall *et al.* 2008; Salgueiro *et al.* 2013).

Senegal

1) *An. arabiensis*
2) *An. brohieri*
3) *An. brunnipes*
4) *An. coluzzii*
5) *An. coustani*
6) *An. domicolus*
7) *An. flavicosta*
8) *An. freetownensis*
9) *An. funestus*
10) *An. gambiae s.s.*
11) *An. hancocki*
12) *An. leesoni*
13) *An. maculipalpis*
14) *An. melas*
15) *An. nili*
16) *An. paludis*
17) *An. pharoensis*
18) *An. pretoriensis*
19) *An. rhodesiensis*
20) *An. rivulorum*
21) *An. rufipes*
22) *An. sergentii*
23) *An. squamosus*
24) *An. wellcomei*
25) *An. ziemanni*

Comment

1) Kyalo *et al.* (2017) did not include *An. leesoni* and *An. rivulorum*, however, Kobylinski (2011) noted their presence near Kedougou, and they are added to the list.

Sierra Leone

1) *An. barberellus*
2) *An. brohieri*
3) *An. brunnipes*
4) *An. coluzzii*
5) *An. coustani*
6) *An. domicolus*
7) *An. flavicosta*
8) *An. freetownensis*
9) *An. funestus*
10) *An. gambiae s.s.*
11) *An. hancocki*
12) *An. hargreavesi*
13) *An. marshallii*
14) *An. melas*
15) *An. moucheti*
16) *An. nili*
17) *An. obscurus*
18) *An. paludis*
19) *An. pharoensis*
20) *An. rhodesiensis*
21) *An. rufipes*
22) *An. smithii*
23) *An. somalicus*
24) *An. squamosus*
25) *An. tenebrosus*
26) *An. theileri*
27) *An. ziemanni*

Comments

1) The only reference in the spreadsheet that Kyalo *et al.* (2017) use for *An. quadriannulatus* in Sierra Leone is Evans (1931), who mentioned *An. quadriannulatus* in passing. However, this publication is about observations carried out in southern Nigeria and should not be included in the Sierra Leone references. *Anopheles quadriannulatus* is removed from the list.
2) No record of *An. cinctus* having been collected in Sierra Leone was found, so it has been removed from the list.

Somalia

1) *An. arabiensis*
2) *An. azaniae*
3) *An. cinereus*
4) *An. coluzzii*
5) *An. coustani*
6) *An. culicifacies*
7) *An. daudi*
8) *An. demeilloni*
9) *An. dthali*
10) *An. funestus*

11) *An. gambiae s.s.*
12) *An. garnhami*
13) *An. merus*
14) *An. nili*
15) *An. paludis*
16) *An. pharoensis*
17) *An. pretoriensis*
18) *An. rhodesiensis*
19) *An. salbaii*
20) *An. sergentii*
21) *An. somalicus*
22) *An. squamosus*
23) *An. tenebrosus*
24) *An. turkhudi*

Comments

1) Kyalo *et al.* (2017) list *An. culicifacies* and *An. sergentii macmahoni*. Diallo *et al.* (2014) reported the presence of *An. culicifacies*. *Anopheles sergentii macmahoni* is a subspecies of *An. sergentii*, so is not included on the list.
2) *Anopheles tenebrosus* was found by Coluzzi (1958) and is added to the list.
3) Diallo *et al.* (2014) reported the presence of *An. garnhami*, which is added to the list.

South Africa

1) *An. arabiensis*
2) *An. ardensis*
3) *An. argenteolobatus*
4) *An. azevedoi*
5) *An. cameroni*
6) *An. carteri*
7) *An. cinctus*
8) *An. cinereus*
9) *An. confusus*
10) *An. coustani*
11) *An. crypticus*
12) *An. cydippis*
13) *An. demeilloni*
14) *An. flavicosta*
15) *An. funestus*
16) *An. gambiae s.s.*
17) *An. garnhami*
18) *An. hughi*
19) *An. implexus*
20) *An. kosiensis*
21) *An. leesoni*
22) *An. letabensis*
23) *An. listeri*
24) *An. longipalpis*
25) *An. maculipalpis*
26) *An. marshallii*
27) *An. merus*
28) *An. mousinhoi*
29) *An. natalensis*

30) *An. nili*
31) *An. parensis*
32) *An. pharoensis*
33) *An. pretoriensis*
34) *An. quadriannulatus*
35) *An. rhodesiensis*
36) *An. rivulorum*
37) *An. rivulorum*-like
38) *An. ruarinus*
39) *An. rufipes*
40) *An. schwetzi*
41) *An. squamosus*
42) *An. tenebrosus*
43) *An. theileri*
44) *An. vaneedeni*
45) *An. vernus*
46) *An. ziemanni*

Comments

1) Kyalo *et al.* (2017) list *An. azevedoi*, *An. kosiensis*, *An. mousinhoi* and *An. turkhudi*. *Anopheles azevedoi* was collected in the Cape Province and initially described as *An. deaconi* by de Meillon & van Eeden (1976). *Anopheles kosiensis* was described from specimens collected in KwaZulu/Natal Province (Coetzee *et al.* 1987). Hunt & Coetzee (1992) noted the presence of *An. mousinhoi* in South Africa. Van der Linde *et al.* (1982) noted the presence of *An. turkhudi* in the Free State Province. Van der Linde *et al.* give three different species identifications for the same specimens (*An. cinereus, An. listeri* and *An. turkhudi*) but the authors stated that they considered their specimens as belonging to the "cinereus group". The former two species both occur in South Africa. Since *An. turkhudi* is confined to the northeastern Horn of Africa, it is unlikely that it occurs in South Africa and is therefore deleted from the list.

2) *Anopheles coustani* is a common species and is added to the list (Gillies & de Meillon 1968; Gillies & Coetzee 1987).

3) Smith *et al.* (1977) recorded *An. flavicosta* from South Africa and this species is added to the list.

4) There is a single record (Miles 1978) of *An. gambiae s.s.* from Pelindaba in northern Kwazulu/Natal Province on the Mozambique border.

5) Mouatcho *et al.* (2018) recorded *An. rivulorum*-like, an as yet unnamed new species.

6) Kyalo *et al.* (2017) also did not include *An. fontinalis*, *An. schwetzi* and *An. theileri.* All three species are mentioned in Gillies & de Meillon (1968) and are included here.

7) Two specimens of *An. schwetzi* from Olifantsvlei, Johannesburg are in the Natural History Museum, London.

South Sudan

1) *An. arabiensis*
2) *An. brohieri*
3) *An. coustani*
4) *An. demeilloni*
5) *An. flavicosta*
6) *An. funestus*
7) *An. gambiae s.l.*
8) *An. garnhami*
9) *An. gibbinsi*
10) *An. implexus*
11) *An. leesoni*
12) *An. longipalpis*
13) *An. maculipalpis*
14) *An. marshallii*

15) *An. moucheti*
16) *An. nili*
17) *An. obscurus*
18) *An. paludis*
19) *An. pharoensis*
20) *An. pretoriensis*
21) *An. rhodesiensis*
22) *An. rivulorum*
23) *An. rufipes*
24) *An. sergentii*
25) *An. squamosus*
26) *An. symesi*
27) *An. wellcomei*
28) *An. ziemanni*

Comment

1) Kyalo *et al.* (2017) did not include *An. gambiae s.s.* Asma (2012) reported *An. gambiae* separately from *An. arabiensis*, but did not distinguish between *An. gambiae s.s*. or *An. coluzzii* (at that time S and M forms), so *An. gambiae s.l.* is noted here. Kyalo *et al.* (2017) also did not include *An. gibbinsi,* which was recorded by Lewis (1956) as *An. marshallii* var. *gibbinsi. Anopheles gibbinsi* is thus added to the list.

Sudan

1) *An. arabiensis*
2) *An. cinereus*
3) *An. coustani*
4) *An. dthali*
5) *An. funestus*
6) *An. gambiae s.s.*
7) *An. implexus*
8) *An. leesoni*
9) *An. maculipalpis*
10) *An. marshallii*
11) *An. multicolor*
12) *An. nili*
13) *An. paludis*
14) *An. pharoensis*
15) *An. pretoriensis*
16) *An. rhodesiensis*
17) *An. rivulorum*
18) *An. rufipes*
19) *An. sergentii*
20) *An. squamosus*
21) *An. turkhudi*
22) *An. wellcomei*
23) *An. ziemanni*

Comments

1) Kyalo *et al.* (2017) list *An. rhodesiensis rupicolus*, which is a subspecies and deleted from the list.
2) Kyalo *et al.* (2017) did not include *An. gambiae s.s.* although it is listed in the database. Petrarca *et al.* (2000) noted a single *An. gambiae* specimen from Sennar. All other specimens in this paper are recorded as the Savannah/Forest chromosomal forms, i.e. *An. gambiae s.s.*, albeit from South Sudan. This species is included in the list here.

3) Lewis (1944) recorded *An. multicolor* from Faras West, an area that was flooded with the creation of Lake Nasser. Later records also place it near Port Sudan (Lewis 1956).
4) There is a single specimen of *An. implexus* in the Natural History Museum, London.

Tanzania (Mainland)

1) *An. arabiensis*
2) *An. ardensis*
3) *An. argenteolobatus*
4) *An. brunnipes*
5) *An. christyi*
6) *An. cinereus*
7) *An. confusus*
8) *An. coustani*
9) *An. cydippis*
10) *An. demeilloni*
11) *An. distinctus*
12) *An. erepens*
13) *An. funestus*
14) *An. gambiae s.s.*
15) *An. garnhami*
16) *An. gibbinsi*
17) *An. implexus*
18) *An. keniensis*
19) *An. kingi*
20) *An. leesoni*
21) *An. letabensis*
22) *An. longipalpis*
23) *An. lovettae*
24) *An. machardyi*
25) *An. maculipalpis*
26) *An. marshallii*
27) *An. merus*
28) *An. moucheti*
29) *An. namibiensis*
30) *An. natalensis*
31) *An. nili*
32) *An. njombiensis*
33) *An. paludis*
34) *An. parensis*
35) *An. pharoensis*
36) *An. pretoriensis*
37) *An. quadriannulatus*
38) *An. rhodesiensis*
39) *An. rivulorum*
40) *An. rufipes*
41) *An. schwetzi*
42) *An. seydeli*
43) *An. squamosus*
44) *An. swahilicus*
45) *An. tenebrosus*
46) *An. theileri*

47) *An. walravensi*
48) *An. wellcomei*
49) *An. wilsoni*
50) *An. ziemanni*

Comments

1) Kyalo *et al.* (2017) included *An. vaneedeni* but this species was not found in the records listed in the database and is removed from the list. In Koekemoer *et al*. (1999), *An. vaneedeni* is reported from Tzaneen, South Africa, which may be the source of confusion. Kyalo *et al.* (2017) also included *An. quadriannulatus*, reported by Fornadel & Norris (2015), and it remains on the list.

2) Bødker *et al.* (2003) reported the collection of 36 *An. letabensis* from the Usambara. It is added to the list.

3) *Anopheles erepens* is noted by Gillies (1958), as *An. wellcomei erepens*, raised to species level by Gillies & Coetzee (1987), and is added to the list.

4) Peters (1953) recorded *An. walravensi* from Njombe, and is added to the list.

5) Wilson (1936) noted *An. moucheti* from Amani, and is added to the list.

6) The IRD database lists *An. namibiensis* from Mahongo, Tanzania.

Tanzania (Zanzibar)

1) *An. arabiensis*
2) *An. aruni*
3) *An. coustani*
4) *An. funestus*
5) *An. gambiae s.s.*
6) *An. leesoni*
7) *An. longipalpis*
8) *An. maculipalpis*
9) *An. marshallii*
10) *An. merus*
11) *An. obscurus*
12) *An. paludis*
13) *An. parensis*
14) *An. pretoriensis*
15) *An. quadriannulatus*
16) *An. rivulorum*
17) *An. rufipes*
18) *An. squamosus*
19) *An. swahilicus*
20) *An. tenebrosus*
21) *An. wellcomei*
22) *An. ziemanni*

Comments

1) Kyalo *et al.* (2017) did not include *An. rufipes* although Gillies & de Meillon (1968) do. It is added to the list.

2) *Anopheles gambiae s.s.* is noted by Mnzava & Kilama (1986) and added to the list.

Togo

1) *An. arabiensis*
2) *An. coluzzii*
3) *An. coustani*
4) *An. flavicosta*

5) *An. funestus*
6) *An. gambiae s.s.*
7) *An. hargreavesi*
8) *An. marshallii*
9) *An. melas*
10) *An. nili*
11) *An. pharoensis*
12) *An, pretoriensis*
13) *An. rivulorum*
14) *An. rufipes*
15) *An. squamosus*
16) *An. wellcomei*
17) *An. ziemanni*

Comments

1) Kyalo *et al.* (2017) did not include *An. domicolus*, *An. leesoni* and *An. smithii* but these are listed in the "Togo and Dahomey" country list of Gillies & de Meillon (1968). They are not included here until such time as their presence in Togo can be confirmed.

2) Kyalo *et al.* (2017) included *An. brunnipes*, *An. maculipalpis*, *An. obscurus*, *An. paludis*, *An. rhodesiensis*, *An. rivulorum* and *An. ziemanni*, but no records were found for these species. Until we have specific records for the presence of these species in Togo, they are removed from the list.

Uganda

1) *An. arabiensis*
2) *An. ardensis*
3) *An. brohieri*
4) *An. bwambae*
5) *An. christyi*
6) *An. cinereus*
7) *An. coustani*
8) *An. cydippis*
9) *An. demeilloni*
10) *An. domicolus*
11) *An. funestus*
12) *An. gambiae s.s.*
13) *An. garnhami*
14) *An. gibbinsi*
15) *An. hancocki*
16) *An. hargreavesi*
17) *An. harperi*
18) *An. implexus*
19) *An. keniensis*
20) *An. kingi*
21) *An. leesoni*
22) *An. longipalpis*
23) *An. maculipalpis*
24) *An. marshallii*
25) *An. moucheti*
26) *An. natalensis*
27) *An. nili*
28) *An. obscurus*

29) *An. paludis*
30) *An. parensis*
31) *An. pharoensis*
32) *An. pretoriensis*
33) *An. quadriannulatus*
34) *An. rhodesiensis*
35) *An. rivulorum*
36) *An. rufipes*
37) *An. squamosus*
38) *An. symesi*
39) *An. tenebrosus*
40) *An. theileri*
41) *An. vinckei*
42) *An. wellcomei*
43) *An. ziemanni*

Comments

1) Mutebi *et al.* (2018) recorded *An. theileri*, which is added to the list.

2) The reference for *An. bervoetsi* provided by Antonio-Nkondjio *et al.* (2008) does not include collection data for this species in Uganda, instead, it refers to collections made in the Demacratic Republic of the Congo. This species is removed from the list.

3) Gibbins (1936) reported a collection of a single specimen of "*costalis* var. *quadriannulatus*", which was confirmed by Alwen Evans. It is added to the list but requires molecular confirmation of species identity.

Zambia

1) *An. arabiensis*
2) *An. argenteolobatus*
3) *An. brunnipes*
4) *An. coustani*
5) *An. cydippis*
6) *An. demeilloni*
7) *An. distinctus*
8) *An. domicolus*
9) *An. funestus*
10) *An. gambiae s.s.*
11) *An. implexus*
12) *An. leesoni*
13) *An. longipalpis*
14) *An. maculipalpis*
15) *An. marshallii*
16) *An. merus*
17) *An. nili*
18) *An. parensis*
19) *An. pharoensis*
20) *An. pretoriensis*
21) *An. quadriannulatus*
22) *An. rhodesiensis*
23) *An. rivulorum*
24) *An. rufipes*
25) *An. schwetzi*
26) *An. seydeli*
27) *An. squamosus*

28) *An. tenebrosus*
29) *An. theileri*
30) *An. vaneedeni*
31) *An. walravensi*
32) *An. wellcomei*
33) *An. ziemanni*

Comments

1) McCullough & Friis-Hansen (1961) noted *An. squamosus cydippis*, which is added to the list as *An. cydippis*.
2) The reference provided to justify the presence of *An. coluzzii* in Zambia (Das *et al.* 2016) does not provide records of this species, so it is removed from the list.
3) *Anopheles tenebrosus* seems to be recognized in Zambia by Gillies & de Meillon (1968), and a specimen from Livingstone is in the Natural History Museum, London.
4) Specimens of *An. merus* from *Avicennia* swamps in Pemba are in the Natural History Museum, London.

Zimbabwe

1) *An. arabiensis*
2) *An. ardensis*
3) *An. argenteolobatus*
4) *An. brunnipes*
5) *An. carteri*
6) *An. cinereus*
7) *An. coluzzii*
8) *An. confusus*
9) *An. coustani*
10) *An. cydippis*
11) *An. demeilloni*
12) *An. distinctus*
13) *An. domicolus*
14) *An. funestus*
15) *An. fuscivenosus*
16) *An. gambiae s.s.*
17) *An. garnhami*
18) *An. keniensis*
19) *An. leesoni*
20) *An. listeri*
21) *An. longipalpis*
22) *An. maculipalpis*
23) *An. marshallii*
24) *An. merus*
25) *An. mousinhoi*
26) *An. multicinctus*
27) *An. natalensis*
28) *An. nili*
29) *An. parensis*
30) *An. pharoensis*
31) *An. pretoriensis*
32) *An. quadriannulatus*
33) *An. rhodesiensis*
34) *An. rivulorum*
35) *An. ruarinus*

36) *An. rufipes*
37) *An. schwetzi*
38) *An. seydeli*
39) *An. squamosus*
40) *An. tenebrosus*
41) *An. theileri*
42) *An. vaneedeni*
43) *An. walravensi*
44) *An. wellcomei*
45) *An. ziemanni*

Comments

1) Reid & Woods (1957) noted *An. multicinctus* (as *An. natalensis* var. *multicinctus*), *An. wellcomei* (as *An. walravensi* var. *milesi*) and *An. distinctus*, and these species are added to the list.

2) Masendu *et al.* (2004) recorded both *An. gambiae s.s.* and *An. coluzzii* in the Zambezi Valley, Zimbabwe. This record of *An. coluzzii* is very unusual, being far out of the known distribution of the species, and needs confirmation. It is retained on the list.

3) Specimens of *An. keniensis* were found in the collection of the Natural History Museum, London, with the locality listed as "Nyangura River, Ndonga"
